# Psychological Impacts and Implications of Aging During a Pandemic

**DOI:** 10.1093/geroni/igab046.2085

**Published:** 2021-12-17

**Authors:** Joseph Mikels, Laura Carstensen, Susan Charles

## Abstract

Despite numerous losses associated with advanced age, older adults typically fare better than their younger counterparts in terms of psychological well-being. However, the COVID-19 pandemic has disproportionately threatened the physical and mental well-being of older adults. How have older versus younger adults been doing? The goal of our symposium is to shed light on this question though presentations of intriguing research findings regarding the psychological impacts of the pandemic on older adults. Stone and Mak will describe their work examining momentary changes in affect, activities, locations, and social interactions over time during the first several months of the pandemic for older individuals. Mikels and colleagues will report on completed and ongoing work illuminating the complex ways in which certain older adults have been faring well during the pandemic, whereas others not so much, with attention to underlying factors. Jeste will discuss a diverse line of research that has examined the relationships between loneliness, social isolation, and compassion in older adults before and during the pandemic. Chi and Carstensen will report on completed and ongoing research that links work and prosocial behavior to wellbeing with consideration of associated age differences. Collectively, these presentations will describe the complex and multifaceted psychological impact that the COVID-19 pandemic has had on older individuals, revealing the multiple ways in which they are resilient as well as vulnerable.

